# Multiple organ scoring systems for predicting in-hospital mortality of sepsis patients in the intensive care unit

**DOI:** 10.1515/med-2025-1229

**Published:** 2025-11-21

**Authors:** Xuan Zhou, Zhenen Zhang, Huimin Wang, Pengfei Chen

**Affiliations:** Department of Intensive Care Unit, Jianhu County Branch of Northern Jiangsu People’s Hospital, Yancheng, Jiangsu, 224700, China; Department of Intensive Care Unit, Jianhu County Branch of Northern Jiangsu People’s Hospital, Jianhu County South Circular Road No. 666 Yancheng, Jiangsu, 224700, China

**Keywords:** sepsis, CCI, LODS, APS III, SAPS II, SOFA, ICU, in-hospital mortality

## Abstract

**Objective:**

This study aims to comprehensively evaluate the Charlson Comorbidity Index (CCI), Logistic Organ Dysfunction System (LODS) score, Acute Physiology Score III (APS III), Simplified Acute Physiology Score II (SAPS II), and Sequential Organ Failure Assessment (SOFA) in order to provide a more scientifically rigorous and effective tool for predicting mortality risk among sepsis patients in the Intensive Care Unit (ICU).

**Methods:**

The demographic information and sepsis-related data were extracted from the MIMIC-IV database for patients admitted to the ICU with a diagnosis of sepsis. The predictive performance of CCI, LODS, APS III, SAPS II, and SOFA scoring systems in terms of ICU mortality was evaluated by comparing receiver operating characteristic (ROC) curves. Multivariate regression identified predictors, later validated with a nomogram. Finally, the dataset was divided into a training set and a validation set at a ratio of 7:3 to assess the clinical utility of the prediction model through ROC curves.

**Results:**

The study enrolled a total of 17,226 patients with a median age of 67.65 (55.79, 78.82) years; among them, males accounted for 57.69%. Within this cohort, a total of 1,115 (6.47%) individuals succumbed during their admission to the ICU. The ROC curve demonstrated that both APS III and LODS score exhibited robust predictive value for ICU mortality. Multivariate regression analysis revealed that CCI, APS III score, LODS score, respiratory rate, body temperature, and race served as potential predictors. Combining these variables into a nomogram showed strong clinical utility, with AUCs of 0.803 (training set) and 0.797 (validation set).

**Conclusions:**

Organ failure scores hold significant clinical relevance in predicting mortality among sepsis patients in the ICU. Augmenting predictive accuracy can be achieved by integrating the CCI, APS III score, LODS score, mean respiratory rate, and body temperature during the initial 24 h following ICU admission.

## 1 Introduction

Sepsis arises from an exaggerated immune response to infection, leading to tissue damage, organ dysfunction, and systemic inflammation [1]. The incidence and mortality rates of sepsis are increasing, presenting a significant challenge to global public health [2]. Sepsis accounts for 30–50% of Intensive Care Unit (ICU) fatalities, making it a leading cause of death in critical care [3,4,5,6].

In light of the significant mortality rate associated with sepsis, timely and accurate assessment of patients’ condition and risk of death is crucial for improving survival rates [7]. In clinical practice, physicians commonly utilize various scoring systems to evaluate the severity and prognosis of septic patients. These scoring systems include, but are not limited to, the Charlson Comorbidity Index (CCI) [8,9], Logistic Organ Dysfunction System (LODS) [10], Acute Physiology Score III (APS III) [11], Simplified Acute Physiology Score II (SAPS II) [12,13], and Sequential Organ Failure Assessment (SOFA) scoring systems [14]. Scoring systems, serving as a pivotal tool in quantifying the severity of a patient’s condition, assume an indispensable role in critical care medicine by employing physiological parameters, laboratory indexes, and other relevant clinical indicators to calculate a numerical value that predicts the patient’s risk of mortality [15,16,17]. Scoring systems prioritize different clinical aspects [18]. For example, the CCI primarily evaluates patients’ comorbidities [19], while the LODS and APS III focus more on the physiological condition of the patient [18,20]. In contrast, SAPS II and SOFA incorporate broader clinical parameters [20,21].

However, despite the pivotal role played by these scoring systems in predicting patient outcomes in sepsis, disparities exist in their respective levels of accuracy. Moreover, due to the intricate and dynamic nature of sepsis patients’ conditions, a singular scoring system often encounters challenges when attempting to comprehensively and accurately reflect their true state [22]. Consequently, the selection and utilization of these scoring systems as well as the integration of multiple system results have emerged as prominent issues within current clinical research for more precise assessment of mortality risk in sepsis patients. Recent studies have highlighted the prognostic value of various inflammatory markers in ICU patients. Mean platelet volume reflects platelet activation and correlates with disease severity and mortality [23]. The neutrophil-to-lymphocyte ratio serves as a marker of systemic inflammation linked to poor outcomes [24]. C-reactive protein (CRP), an acute-phase protein, is associated with inflammation and mortality risk [25]. Platelet distribution width, which measures platelet size variation, is linked to thrombotic risk and inflammation [26]. Despite the potential of these markers, existing scoring systems for predicting ICU mortality do not fully integrate them.

In conclusion, the objective of this study was to elucidate the prognostic value of each scoring system in assessing mortality risk among sepsis patients during their ICU stay. By conducting a comprehensive evaluation of the CCI, LODS, APS III, SAPS II, and SOFA scoring systems, while considering vital signs monitoring alongside multiple scoring systems, our aim is to provide a more scientifically robust and effective clinical prediction tool for evaluating the risk of mortality in sepsis.

## 2 Methods and methods

### 2.1 Study design

The data for this study were extracted from the Medical Information Mart for Intensive Care-IV (MIMIC-IV) database, encompassing comprehensive clinical information on a cohort of more than 190,000 patients and 450,000 hospitalizations admitted to Beth Israel Deaconess Medical Center (BIDMC) between 2008 and 2019 [24]. This extensive database comprises patient demographic details, laboratory test results, medication records, vital signs measurements, surgical procedures performed, disease diagnosis, drug management specifics, follow-up survival status updates, as well as other pertinent information. This study presents an analysis of a publicly available database, for which no approval from the Institutional Review Board of Jianhu County branch of Northern Jiangsu People’s Hospital was deemed necessary.

Patients admitted to the ICU for the first time with a diagnosis of sepsis were included with information from MIMIC-IV. The inclusion criteria were as follows: (1) Over 18 years of age and (2) diagnosis with sepsis. The exclusion criteria were defined as follows: patients who (1) with previous history of hospitalization, (2) duration of the ICU stay less than 24 h, and (3) with malignant tumors.

### 2.2 Date extraction

The diagnosis of sepsis was made in accordance with the sepsis-3 criteria [25]. The demographic information, encompassing age, gender, ethnicity, and body mass index (BMI), was extracted from the database utilizing Navicate Premium 16.0. The CCI, LODS, SOFA, APS III, SAPS II, GCS scores, and laboratory parameters including creatine were evaluated 24 h post admission to the ICU. For variables with multiple measurements, the mean value was used.

A patient was considered to have expired in the ICU if their time of death coincided with their discharge time from the ICU. If these times coincided, it was concluded that the patient deceased during their stay in the ICU; otherwise, they were considered to be alive.

### 2.3 Statistical analysis

Measurement data that followed a normal distribution were presented as Mean ± SD. For data with equal variances, a *T*-test was utilized to compare two groups. In cases where the measurement data did not conform to a normal distribution, quartiles and median values were used instead, and differences between any two groups were compared using the Mann–Whitney test. Count data were expressed in terms of frequencies and proportions, with group differences assessed through chi-square testing. Statistical significance was established when *P* < 0.05.

The predictive efficacy of each scoring system for ICU sepsis patients’ mortality was assessed using receiver operating characteristic (ROC) curve analysis. Potential predictors, including vital signs, laboratory parameters, and scores, were identified through regression analysis. The variables exhibiting a significance level of *P* < 0.05 in the univariate regression analysis were incorporated into the multivariate regression analysis and examined using the two-way stepwise regression method. A nomogram was employed to present the final combined score and baseline characteristics of the patients. Furthermore, a training set and validation set were created by dividing the dataset in a 7:3 ratio. The performance of the final model was validated using ROC curve analysis.


**Ethical approval:** This study is an analysis of a public database. Approval from the Institutional Review Board was not required.
**Consent for publication:** Not applicable.

## 3 Results

### 3.1 Clinical characteristics of patients with sepsis

**Figure 1:**
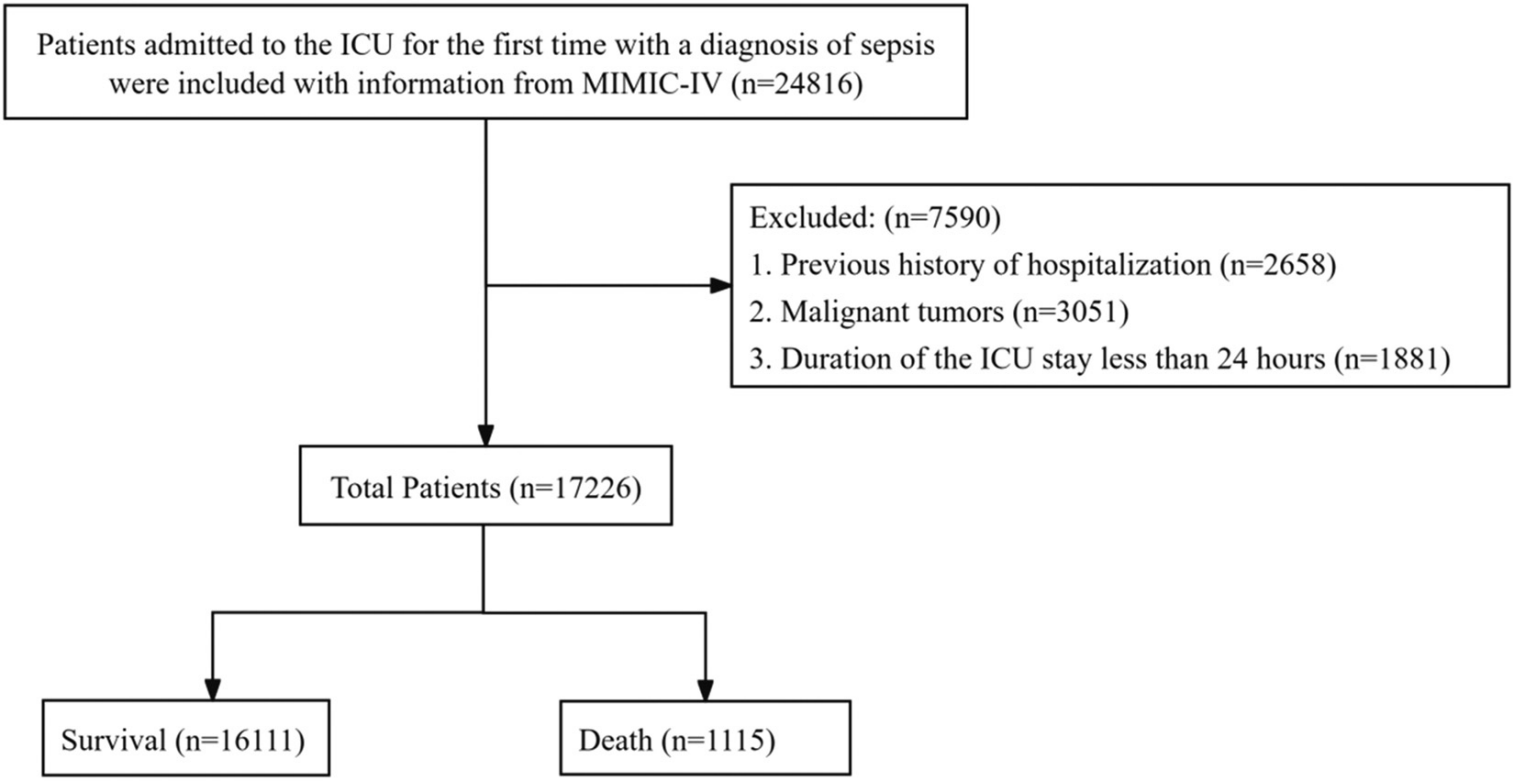
The process of information extraction and the screening criteria for sepsis patients.

The process of data extraction and screening was carried out following the workflow illustrated in Figure 1. A total of 24,816 adult patients were diagnosed with sepsis and admitted to the hospital for their initial presentation. After excluding 2,658 patients with a history of prior hospitalization, 3,051 patients with malignancy, and 1,881 patients who stayed in the ICU for less than 24 h, a total of 17,226 eligible patients were enrolled. Among them, 1,115 patients (6.47%) succumbed during their stay in the ICU. The enrolled patients had a median age of 67.65 (55.79, 78.82) years, with males accounting for 57.69% of the cohort. The baseline characteristics of the enrolled patients are presented in Table 1.

**Table 1:** Baseline data of the enrolled patients

Variables	Total (*n* = 17,226)	Survival (*n* = 16,111)	Death (*n* = 1,115)	Statistic	*P*
Age, Years, M (*Q*_1_, *Q*_3_)	67.65 (55.79, 78.82)	67.51 (55.69, 78.64)	70.16 (57.23, 81.64)	*Z* = −4.29	<0.001
Gender, *n* (%)				*χ* ^2^ = 4.59	0.032
Female	7,289 (42.31)	6,783 (42.10)	506 (45.38)		
Male	9,937 (57.69)	9,328 (57.90)	609 (54.62)		
Race, *n* (%)				*χ* ^2^ = 236.70	<0.001
Asian	438 (2.54)	411 (2.55)	27 (2.42)		
Black	1,306 (7.58)	1,229 (7.63)	77 (6.91)		
White	11,450 (66.47)	10,867 (67.45)	583 (52.29)		
Hispanic/Latino	550 (3.19)	518 (3.22)	32 (2.87)		
Other	744 (4.32)	706 (4.38)	38 (3.41)		
Unknown	2,738 (15.89)	2,380 (14.77)	358 (32.11)		
BMI, kg/m^2^, M (*Q*_1_, *Q*_3_)	28.30 (24.60, 33.10)	28.40 (24.60, 33.10)	27.90 (24.13, 32.90)	*Z* = 2.02	0.043
SOFA, score, M (*Q*_1_, *Q*_3_)	3.00 (2.00, 4.00)	3.00 (2.00, 4.00)	4.00 (2.00, 6.00)	*Z* = −12.29	<0.001
CCI, score, M (*Q*_1_, *Q*_3_)	4.00 (3.00, 6.00)	4.00 (3.00, 6.00)	5.00 (3.00, 7.00)	*Z* = −9.43	<0.001
Creatinine, mg/dL, M(*Q*_1_, *Q*_3_)	1.00 (0.70, 1.50)	1.00 (0.70, 1.50)	1.30 (0.90, 2.10)	*Z* = −13.90	<0.001
GCS, score, M (*Q*_1_, *Q*_3_)	15.00 (15.00, 15.00)	15.00 (15.00, 15.00)	15.00 (15.00, 15.00)	*Z* = −0.81	0.415
APS III, score, M (*Q*_1_, *Q*_3_)	45.00 (33.00, 60.00)	44.00 (32.00, 59.00)	66.00 (50.00, 88.00)	*Z* = −28.63	<0.001
LODS, score, M (*Q*_1_, *Q*_3_)	5.00 (3.00, 7.00)	5.00 (3.00, 7.00)	8.00 (6.00, 10.00)	*Z* = −29.01	<0.001
SAPS II, score, M (*Q*_1_, *Q*_3_)	37.00 (30.00, 47.00)	37.00 (29.00, 46.00)	49.00 (39.00, 61.00)	*Z* = −26.20	<0.001
Heart Rate, bpm, M (*Q*_1_, *Q*_3_)	84.62 (75.39, 96.26)	84.42 (75.33, 95.92)	87.68 (76.44, 102.18)	*Z* = −5.71	<0.001
SBP, mmHg, M (*Q*_1_, *Q*_3_)	113.54 (105.64, 124.04)	113.79 (105.90, 124.17)	110.08 (102.32, 122.32)	*Z* = −7.72	<0.001
DBP, mmHg, M (*Q*_1_, *Q*_3_)	60.20 (54.45, 66.95)	60.23 (54.52, 66.85)	59.56 (53.03, 68.30)	*Z* = 1.12	0.262
Respiratory rate, insp/min, M (*Q*_1_, *Q*_3_)	18.80 (16.65, 21.73)	18.68 (16.58, 21.54)	20.78 (18.21, 24.32)	*Z* = −16.17	<0.001
Temperature, °C, M (*Q*_1_, *Q*_3_)	36.87 (36.59, 37.23)	36.87 (36.60, 37.23)	36.76 (36.32, 37.20)	*Z* = 8.40	<0.001
Diabetes with complication, *n* (%)				*χ* ^2^ = 0.18	0.675
No	15,636 (90.77)	14,620 (90.75)	1,016 (91.12)		
Yes	1,590 (9.23)	1,491 (9.25)	99 (8.88)		
Mechanical ventilation, *n* (%)				*χ* ^2^ = 47.05	<0.001
No	5,748 (37.51)	5,454 (38.25)	294 (27.68)		
Yes	9,574 (62.49)	8,806 (61.75)	768 (72.32)		

M: Median; *Q*_1_: 1st Quartile; *Q*_3_: 3rd Quartile. Z: Mann–Whitney test; *χ* ^2^: Chi square test. BMI: Body Mass Index; SOFA: Sequential Organ Failure Assessment; CCI: Charlson Comorbidity Index; GCS: Glasgow Coma Scale; APS III: Acute Physiology Score III; LODS: Logistic Organ Dysfunction Score; SAPS II: Simplified Acute Physiology Score II; SBP: systolic blood pressure; DBP: diastolic blood pressure.

The death group exhibited significantly higher levels of age, CCI, LODS, SOFA, APS III, SAPS II scores, GCS scores, creatine levels, heart rate within 24 h of admission to ICU compared to the survival group (*P* < 0.001). However, the deceased group exhibited a lower BMI and mean body temperature during the initial 24-h period in the ICU compared to the survivor group. Additionally, the proportion of females (*P* = 0.032) and patients receiving mechanical ventilation (*P* < 0.001) in the death group were higher compared to those in the survival group.

### 3.2 The effectiveness of five scoring systems in predicting ICU mortality

**Figure 2:**
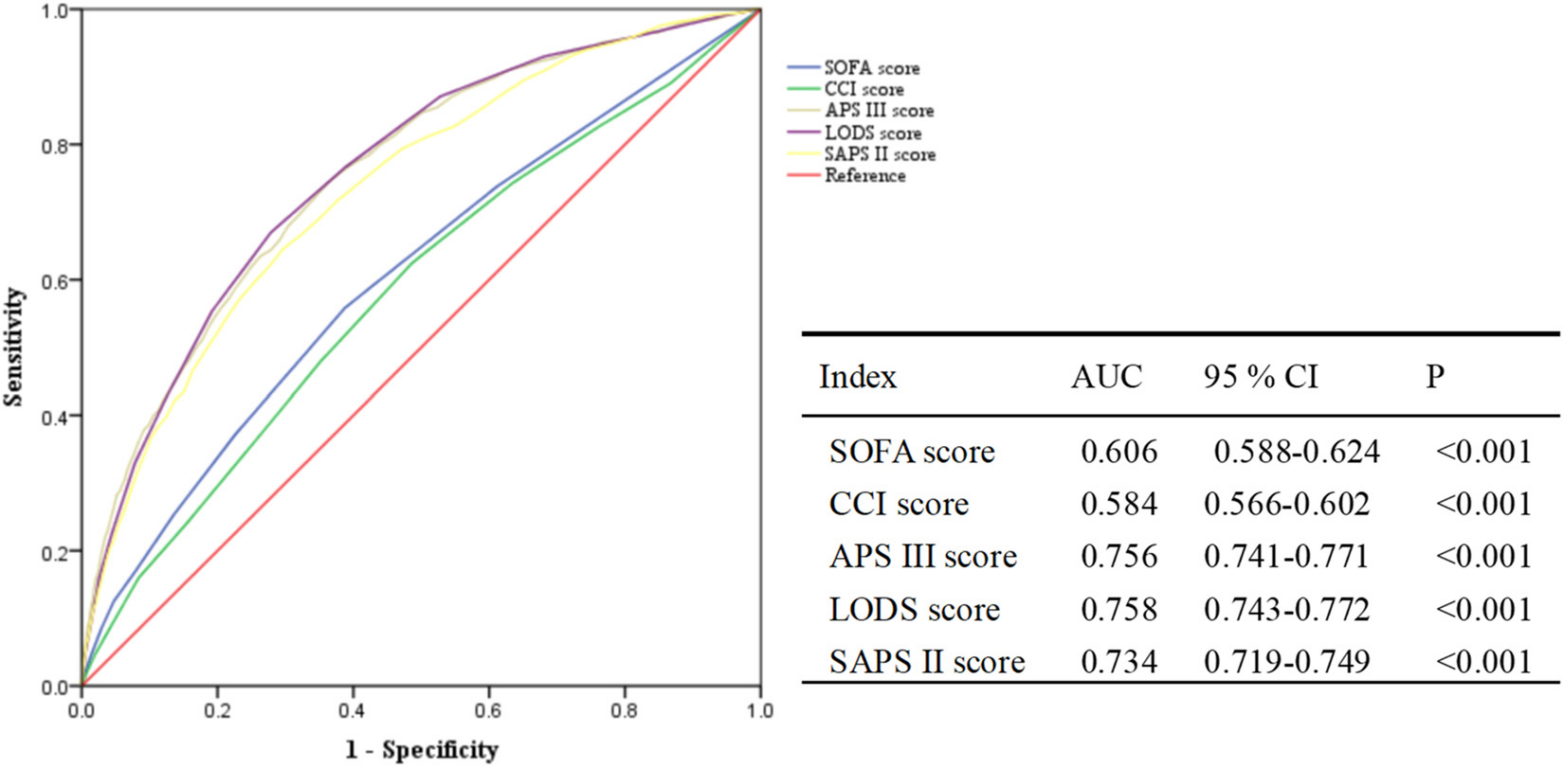
Prediction of mortality in septic ICU patients using multiple scoring systems. SOFA: Sequential Organ Failure Assessment; CCI: Charlson comorbidity index; APS III: Acute Physiology Score III; LODS: Logistic Organ Dysfunction Score; SAPS II: Simplified acute physiology score II; AUC: Area under the receiver operating characteristic curve; CI: confidence interval.

The ROC curve demonstrated that both APS III (AUC: 0.756, *P* < 0.001) and LODS (AUC: 0.758, *P* < 0.001) score exhibited robust predictive value for ICU mortality. The AUC (0.734, *P* < 0.001) of the SAPS II score exhibited a slightly lower value compared to that of the APS III and LODS scores (Figure 2). Additionally, the sensitivity and specificity for each scoring system were as follows: APS III (sensitivity: 76.1%, specificity: 68.4%), LODS (sensitivity: 75.8%, specificity: 69.2%), SAPS II (sensitivity: 71.3%, specificity: 65.7%), SOFA (sensitivity: 67.5%, specificity: 63.9%), and CCI (sensitivity: 59.2%, specificity: 60.1%). Calibration metrics also indicated that APS III and LODS showed better agreement between predicted and observed outcomes compared to the other scoring systems (Table S2).

### 3.3 Regression analysis to identify potential factors

**Figure 3:**
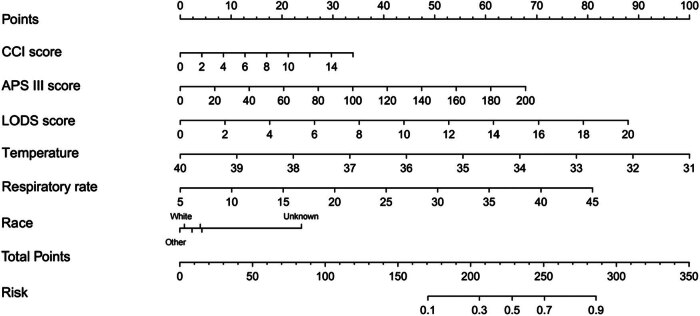
Nomogram for the prediction of death in the ICU. CCI: Charlson Comorbidity Index; APS III: Acute Physiology Score III; LODS: Logistic Organ Dysfunction Score.

The potential predictors identified through univariate and multivariate regression analyses, with ICU mortality as the outcome, are presented in Table 2. Multivariate regression analysis revealed that CCI (OR = 1.10; 95% CI:(1.06, 1.14); *P* < 0.001), APS III score (OR = 1.01; 95% CI:(1.01, 1.02); *P* < 0.001), LODS score (OR = 1.14; 95% CI:(1.09, 1.20); *P* < 0.001), respiratory rate (OR = 1.07; 95% CI:(1.05, 1.10); *P* < 0.001), body temperature (OR = 0.75; 95% CI:(0.67, 0.85); *P* < 0.001), and unknown race (OR = 2.82; 95% CI:(2.27, 3.49); *P* < 0.001) served as potential predictors. The integration of these indicators into a nomogram demonstrated significant clinical relevance (Figure 3).

**Table 2:** Screening of covariates as potential risk factors

Variables	Univariate analysis			Multivariate analysis		
	*β*	OR (95%CI)	*P*	*β*	OR (95%CI)	*P*
Age, Years, M (*Q*_1_, *Q*_3_)	0.01	1.01 (1.01, 1.01)	<0.001			
BMI, kg/m^2^, M (*Q*_1_, *Q*_3_)	−0.01	0.99 (0.98, 1.00)	0.138			
SOFA, score, M (*Q*_1_, *Q*_3_)	0.18	1.20 (1.16, 1.24)	<0.001			
CCI, score, M (*Q*_1_, *Q*_3_)	0.11	1.12 (1.09, 1.15)	<0.001	0.09	1.10 (1.06, 1.14)	<0.001
GCS, score, M (*Q*_1_, *Q*_3_)	0.01	1.01 (0.97, 1.04)	0.739			
APS III, score, M (*Q*_1_, *Q*_3_)	0.04	1.04 (1.03, 1.04)	<0.001	0.01	1.01 (1.01, 1.02)	<0.001
LODS, score, M (*Q*_1_, *Q*_3_)	0.29	1.33 (1.30, 1.36)	<0.001	0.13	1.14 (1.09, 1.20)	<0.001
SAPS II, score, M (*Q*_1_, *Q*_3_)	0.06	1.06 (1.05, 1.06)	<0.001			
Heart Rate, bpm, M (*Q*_1_, *Q*_3_)	0.01	1.01 (1.01, 1.02)	<0.001			
SBP, mmHg, M (*Q*_1_, *Q*_3_)	−0.01	0.99 (0.98, 0.99)	<0.001	0.01	1.01 (1.00, 1.01)	0.102
DBP, mmHg, M (*Q*_1_, *Q*_3_)	−0.00	1.00 (0.99, 1.00)	0.481			
Respiratory rate, insp/min, M (*Q*_1_, *Q*_3_)	0.11	1.12 (1.10, 1.13)	<0.001	0.07	1.07 (1.05, 1.10)	<0.001
Temperature, °C, M (*Q*_1_, *Q*_3_)	−0.55	0.58 (0.52, 0.64)	<0.001	−0.28	0.75 (0.67, 0.85)	<0.001
Creatinine, mg/dL, M (*Q*_1_, *Q*_3_)	0.10	1.11 (1.07, 1.14)	<0.001			
Gender						
Male	Reference					
Female	0.14	1.14 (0.99, 1.33)	0.071			
Race						
White	Reference			Reference		
Unknown	1.06	2.90 (2.45, 3.42)	<0.001	1.04	2.82 (2.27, 3.49)	<0.001
Hispanic/Latino	0.03	1.03 (0.65, 1.64)	0.891	0.21	1.24 (0.70, 2.18)	0.460
Black	0.13	1.14 (0.85, 1.53)	0.395	0.10	1.11 (0.75, 1.63)	0.613
Other	−0.11	0.90 (0.59, 1.36)	0.615	−0.12	0.89 (0.52, 1.51)	0.668
Asian	0.09	1.09 (0.65, 1.82)	0.746	0.04	1.04 (0.51, 2.14)	0.907
Diabetes with complication						
No	Reference					
Yes	−0.06	0.94 (0.73, 1.21)	0.634			
Mechanical ventilation						
Yes	Reference					
No	−0.49	0.61 (0.52, 0.72)	<0.001			

OR: odds ratio, CI: confidence interval. BMI: Body Mass Index; SOFA: Sequential Organ Failure Assessment; CCI: Charlson comorbidity index; GCS: Glasgow Coma Scale; APS III: Acute Physiology Score III; LODS: Logistic Organ Dysfunction Score; SAPS II: Simplified acute physiology score II; SBP: systolic blood pressure; DBP: diastolic blood pressure.

### 3.4 Predictive performance assessed in training and validation sets

The training set comprised 12,058 patients, while the validation set included 5,168 patients. Table S1 presents a comparison of the characteristics between these two sets. The clinical data revealed no statistically significant disparities between the randomly allocated training set and the validation set.

**Figure 4:**
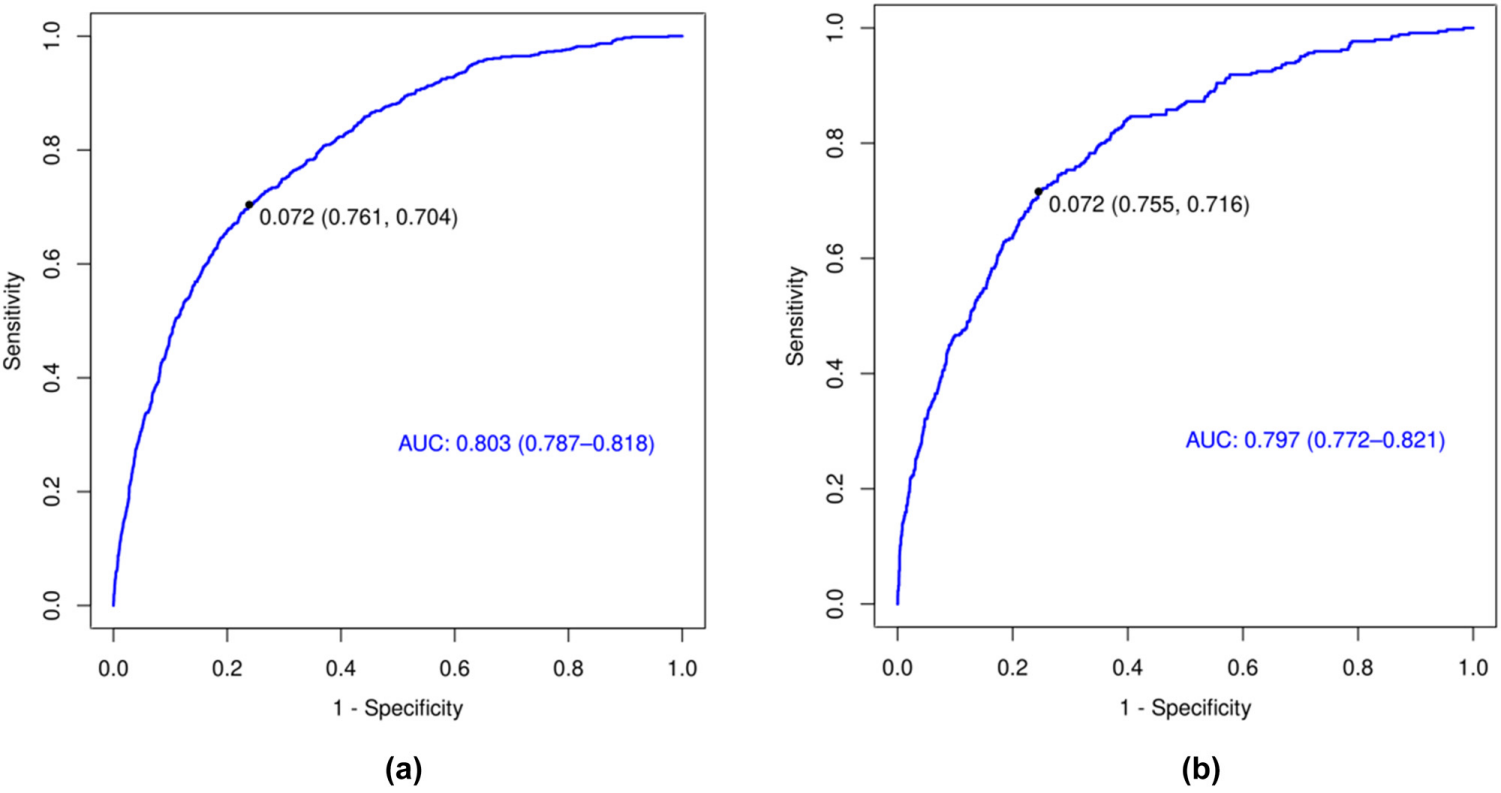
The performance of the prediction model in both the training and validation sets. (a) Evaluation of effectiveness in the training set; (b) assessment of performance in the validation set.

The ROC curves achieved AUCs of 0.803 (training set) and 0.797 (validation set), indicating robust predictive performance. The training set demonstrated predictive sensitivities and specificities of 76.1 and 70.4%, respectively. In the validation set, corresponding sensitivities were observed to be 75.5 and 71.6% (Figure 4).

## 4 Discussion

The accurate prediction of mortality risk in sepsis has become imperative for optimizing patient survival, given its emergence as a predominant and lethal condition within the intensive care unit [26]. The objective of this study was to assess the efficacy of CCI, LODS, APS III, SAPS II, and SOFA scoring systems in predicting mortality among sepsis patients during their stay in the ICU. Retrospective analysis was conducted on clinical data from MIMIC-IV database to compare the predictive accuracy of these scoring systems. The findings of this investigation revealed variations in terms of accuracy among five scoring systems, indicating their prognostic capability for mortality prediction in septic patients. Notably, individual scoring systems such as APS III and LODS demonstrated significant predictive value. However, it is worth noting that a combination comprising CCI, APS III score, LODS score, average respiratory rate, and body temperature within 24 h after ICU admission along with race could further improve the accuracy of mortality risk predictions.

The APS III scoring system incorporates a range of physiological parameters, including blood pressure, body temperature, heart rate, respiratory rate, blood oxygen saturation, blood pH, serum electrolyte levels, and renal function indicators [27]. Each parameter is assigned a distinct score based on its correlation with the risk of mortality. By evaluating the APS III score of patients admitted to the ICU, physicians can promptly determine the severity of their condition and facilitate prognostication as well as evaluation of treatment response [28]. Additionally, the APS III score can serve as a valuable tool for assessing the effectiveness of various therapeutic interventions and guiding adjustments to clinical management plans [29]. The study also yielded consistent findings, demonstrating a remarkable predictive efficacy of a single APS III score in sepsis patients for ICU mortality with an AUC of 0.756.

The LODS scoring system encompasses six major organ systems, namely the respiratory, circulatory, hepatic, coagulation, nervous, and renal systems [30]. Each organ system’s dysfunction is accompanied by a corresponding scoring criterion. By measuring and evaluating the patient’s physiological parameters, the cumulative score can be computed to assess the extent of organ dysfunction [30,31]. The LODS scoring system has been shown to possess exceptional precision and reliability in predicting mortality risk among patients in the ICU, particularly those suffering from sepsis [32]. Similarly, in the present study, the prognostic efficacy of LODS on mortality in ICU septic patients was also substantial and superior to that of other scoring systems. When evaluating mortality in sepsis patients, the area under the curve (AUC) of the LODS score for predicting both 28-day and 90-day prognosis outperformed that of both SOFA and qSOFA scores [33]. Moreover, the admission of sepsis patients to the ICU is associated with an increased risk of in-hospital mortality, as evidenced by a higher LODS score [6].

The CCI scoring system consists of 19 major comorbid conditions, each assigned a weight ranging from 1 to 6 points based on its impact on the risk of mortality [34]. A higher cumulative score for a patient indicates an increased probability of death within the initial year. The CCI scoring system demonstrates certain predictive value in assessing mortality risk among patients [35]. The performance of CCI, however, was not satisfactory when solely evaluating mortality in septic patients during their stay in the ICU, aligning with findings from other studies [36]. The incorporation of CCI with other scoring systems enhances its efficacy in predicting both short-term and long-term mortality [36].

However, in this study, we did not observe a significant association between SAPS II and ICU mortality in septic patients. This may be attributed to the limited inclusion of comprehensive prognostic factors, such as immune and nutritional status of patients, within the current scoring system. Furthermore, potential changes in population demographics and treatment techniques could potentially compromise the predictive accuracy of the SAPS II scoring system, highlighting the necessity for periodic calibration and updates. Additionally, numerous studies have demonstrated that the SAPS II score exhibits a superior predictive value for mortality in ICU patients with non-septic shock compared to LODS [37,38]. The observed discrepancy in this study may be attributed to variations in the included covariates and the scoring system employed, which incorporates APS III and CCI. Zhu et al. employed six scoring systems to predict 28-day mortality in both general and specialized care units, with varying applicability across different types of ICU. For example, APS III or SAPS II can be utilized for predicting 28-day mortality in general ICUs, while the use of APS III or LODS is more effective in cardiovascular ICU for forecasting the risk of 28-day mortality [18]. The accuracy of predicting mortality risk in ICU patients with sepsis further improved by integrating multiple scoring systems with the physiological status of the patients in this study [39–41].

This study systematically compared and analyzed the value and efficacy of CCI, LODS, APS III, SAPS II, and SOFA scoring systems in predicting sepsis patient mortality within ICU settings. The findings demonstrate that although these scoring systems possess their individual strengths in prognosticating death risks among sepsis patients. Moreover, it is worth noting that a combination comprising CCI, APS III score, LODS score, average respiratory rate and body temperature within 24 h after ICU admission along with race could further improve the accuracy of mortality risk predictions. The inclusion of respiratory rate and temperature enhances the model by capturing dynamic physiological derangements not fully represented in static scoring systems like APS III. For example, hypothermia (<36°C) is strongly associated with immunosuppression and multi-organ failure in sepsis [42], while tachypnea may signal early respiratory compromise [43]. The inclusion of race as a predictor in our model requires careful interpretation. While race demonstrated statistical significance in predicting ICU mortality (*P* < 0.05), it is not a direct biological risk factor. Instead, race may serve as a surrogate for socioeconomic status, access to healthcare, or comorbidities that are associated with disparities in patient outcomes. For example, patients from certain racial backgrounds may experience barriers in accessing timely medical care or may have higher burdens of chronic diseases due to social determinants of health. These factors can indirectly influence mortality risk and thus contribute to the predictive power of race in our model. It is crucial to approach the use of race in predictive models with caution and to recognize the potential for confounding by these underlying social and economic factors. Future research should aim to disentangle the specific pathways through which race interacts with healthcare access and quality, socioeconomic status, and comorbidities to affect mortality outcomes. This would not only enhance the precision of predictive models but also inform interventions aimed at reducing healthcare disparities. While our nomogram leverages interpretable logistic regression, future studies should adopt ML techniques to refine variable selection and mitigate redundancy. For instance, LASSO regression could retain only non-overlapping predictors (e.g., APS III or respiratory rate), while tree-based methods (e.g., XGBoost) might identify novel interactions between scores and vital signs. Hybrid models combining ML-optimized features with clinician-friendly interfaces (e.g., SHAP values) could balance accuracy and interpretability. We propose that ML-driven approaches will be critical for advancing sepsis prediction models beyond the limitations of traditional scoring systems.

To translate our findings into practical clinical applications, we recommend integrating the nomogram into existing ICU protocols as a decision-support tool. The nomogram can serve as a valuable aid in identifying patients at high risk of mortality, allowing clinicians to prioritize resources and tailor interventions accordingly. For instance, patients with a predicted high mortality risk based on the nomogram could be monitored more closely, receive earlier intervention with evidence-based sepsis therapies, or be considered for enrollment in clinical trials exploring novel treatment approaches. Additionally, the nomogram can facilitate communication between healthcare providers and families by providing a data-driven prognosis, thereby supporting shared decision-making. It is important to note that while the nomogram offers a quantitative assessment, it should complement – rather than replace – clinical judgment. Future research could explore the implementation of such tools in electronic health record systems to streamline their use in busy ICU settings and further evaluate their impact on patient outcomes. The utilization of scoring systems not only facilitates physicians in making more precise risk assessments, but also provides guidance for clinical decision-making and resource allocation. For patients at high risk, physicians can potentially employ more aggressive treatment and intervention based on the scoring system results, thereby potentially reducing the patient’s mortality risk.

This study has certain limitations. First, the generalizability of our findings may be limited due to the use of data from the MIMIC-IV database, which is derived from a single US hospital system. Differences in patient demographics, such as age, comorbidities, and socioeconomic status, could influence the predictive performance of the scoring systems in other settings. Additionally, variations in sepsis management protocols, including differences in diagnostic criteria, treatment algorithms, and resource availability, may affect the applicability of our results. For example, healthcare systems with limited access to advanced monitoring equipment or specialized ICU care might observe different predictive accuracies for these scoring systems. Furthermore, cultural and regional differences in medical practice could also play a role in how these scoring systems perform in diverse populations. Future research should aim to validate these findings in multi-center datasets from different geographic regions to assess the generalizability of our conclusions across various healthcare settings. Additionally, it is important to consider that variations in adaptability and sensitivity among different scoring systems may exist due to differences in their original design and scoring elements. Furthermore, this study did not extensively explore the performance of these scoring systems in various sepsis subtypes or clinical scenarios. Therefore, future research could focus on comprehensively applying these scoring systems or developing new predictive models to further optimize risk assessment and management for patients with sepsis. The model did not account for dynamic interventions such as vasopressor use or fluid resuscitation, which may modulate mortality risk. For instance, delayed vasopressor initiation is associated with higher sepsis mortality, and fluid overload worsens outcomes in septic shock. Future studies should explore integrating these variables into longitudinal prediction models. Integrating biochemical markers such as lactate, procalcitonin, CRP, and IL-6 with clinical scoring systems could enhance predictive accuracy. These markers provide insights into critical pathological processes in sepsis. Future research should explore combining these markers with clinical data to improve risk stratification and guide clinical decision-making.

## 5 Conclusion

Organ failure scores hold significant clinical relevance in predicting mortality among sepsis patients in the ICU. Augmenting predictive accuracy can be achieved by integrating the CCI, APS III score, LODS score, mean respiratory rate, and body temperature during the initial 24 h following ICU admission.

## Supplementary Material

Supplementary Table
