# Supplementary material for: Multiple organ scoring systems for predicting in-hospital mortality of sepsis patients in the intensive care unit
Source: Open Med (Wars). 2025 Nov 21;20(1):20251229. doi: 10.1515/med-2025-1229 (PMC12658730; doi:10.1515/med-2025-1229)
Supplement: Supplementary Table [file med-2025-1229-sm.pdf]

Supplementary material

Table S1: The comparative analysis between the training set and validation set

Variable	Total (n = 17,226)	Training set (n = 12,058)	Validation set (n = 5,168)	Statistic	P
Age, Years, M (Q_1, Q_3)	67.65 (55.79, 78.82)	67.74 (55.75, 78.99)	67.43 (55.90, 78.45)	Z = 0.735	0.463
BMI, kg/m^2, M (Q_1, Q_3)	28.30 (24.60, 33.10)	28.30 (24.60, 33.10)	28.40 (24.60, 33.00)	Z = 0.139	0.890
SOFA, score, M (Q_1, Q_3)	3.00 (2.00, 4.00)	3.00 (2.00, 4.00)	3.00 (2.00, 4.00)	Z = 1.612	0.120
CCI, score, M (Q_1, Q_3)	4.00 (3.00, 6.00)	4.00 (3.00, 6.00)	4.00 (3.00, 6.00)	Z = 0.459	0.648
SAPS II, score, M (Q_1, Q_3)	37.00 (30.00, 47.00)	37.00 (30.00, 47.00)	37.00 (30.00, 47.00)	Z = 0.618	0.537
APS III, score, M (Q_1, Q_3)	45.00 (33.00, 60.00)	44.00 (33.00, 60.00)	45.00 (33.00, 61.00)	Z = 1.878	0.054
GCS, score, M (Q_1, Q_3)	15.00 (15.00, 15.00)	15.00 (15.00, 15.00)	15.00 (15.00, 15.00)	Z = 1.955	0.165
LODS, score, M (Q_1, Q_3)	5.00 (3.00, 7.00)	5.00 (3.00, 7.00)	5.00 (3.00, 7.00)	Z = 1.311	0.193
Heart Rate, bpm, M (Q_1, Q_3)	84.62 (75.39, 96.26)	84.52 (75.14, 96.18)	84.83 (75.91, 96.45)	Z = 1.376	0.169
Temperature, °C, M (Q_1, Q_3)	36.87 (36.59, 37.23)	36.86 (36.59, 37.23)	36.88 (36.60, 37.24)	Z = 1.858	0.063
Respiratory rate, insp/min, M (Q_1, Q_3)	18.80 (16.65, 21.73)	18.76 (16.61, 21.76)	18.87 (16.72, 21.66)	Z = 0.933	0.351
SBP, mmHg, M (Q_1, Q_3)	113.54 (105.64, 124.04)	113.64 (105.79, 124.09)	113.22 (105.40, 123.90)	Z = 1.740	0.082
DBP, mmHg, M (Q_1, Q_3)	60.20 (54.45, 66.95)	60.29 (54.52, 67.03)	59.97 (54.29, 66.69)	Z = 1.960	0.052
Creatinine, mg/dL, M(Q_1, Q_3)	1.00 (0.70, 1.50)	1.00 (0.70, 1.50)	1.00 (0.80, 1.50)	Z = 0.055	0.956
Gender, n (%)				χ^2 = 0.157	0.692
Female	7289 (42.31)	5114 (42.41)	2175 (42.09)		
Male	9937 (57.69)	6944 (57.59)	2993 (57.91)		
Race, n (%)				χ^2 = 2.452	0.784
Asian	438 (2.54)	312 (2.59)	126 (2.44)		
Black	1306 (7.58)	905 (7.51)	401 (7.76)		
Hispanic/Latino	550 (3.19)	375 (3.11)	175 (3.39)		
Other	744 (4.32)	527 (4.37)	217 (4.20)		
Unknown	2738 (15.89)	1936 (16.06)	802 (15.52)		
White	11450 (66.47)	8003 (66.37)	3447 (66.70)		
Death in the ICU, n (%)				χ^2 = 1.241	0.265
No	16111 (93.53)	11294 (93.66)	4817 (93.21)		
Yes	1115 (6.47)	764 (6.34)	351 (6.79)		
Mechanical ventilation, n (%)				χ^2 = 1.437	0.231
No	5748 (37.51)	4056 (37.82)	1692 (36.80)		
Yes	9574 (62.49)	6668 (62.18)	2906 (63.20)		

M: Median; Q_1: 1st Quartile; Q_3: 3st Quartile. Z: Mann,Whitney test; χ^2: Chi,square test. Abbreviation: BMI: Body Mass Index; SOFA: Sequential Organ Failure Assessment; CCI: Charlson comorbidity index; SAPS II: Simplified acute physiology score II; APS III: Acute Physiology Score III; GCS: Glasgow Coma Scale; LODS: Logistic Organ Dysfunction Score; SBP: systolic blood pressure; DBP: diastolic blood pressure.

Table S2: The sensitivity, specificity, and calibration metrics for all scoring systems

Scoring System	AUC (95% CI)	Sensitivity (%)	Specificity (%)	Hosmer-Lemeshow Test (*p*-value)
APS III	0.756 (0.732–0.780)	76.1	68.4	0.12
LODS	0.758 (0.734–0.782)	75.8	69.2	0.09
SAPS II	0.734 (0.710–0.758)	71.3	65.7	0.03*
SOFA	0.698 (0.673–0.723)	67.5	63.9	0.01*
CCI	0.621 (0.594–0.648)	59.2	60.1	<0.001*

A p-value < 0.05 in the Hosmer-Lemeshow test indicates poor calibration.
